# HIV/AIDS and home-based health care

**DOI:** 10.1186/1475-9276-7-8

**Published:** 2008-03-18

**Authors:** Pamella A Opiyo, Takashi Yamano, TS Jayne

**Affiliations:** 1 Netherlands Development Organisation (SNV), Lunzuwa Rd., P.O Box 410576, Kasama, Zambia; 2 The Foundation for Advanced Studies on International Development in Tokyo, Japan; 3 International Development, Department of Agriculture, Food, and Resource Economics, 216a Agriculture Hall Michigan State University, East Lansing, Michigan 48824, USA

## Abstract

This paper highlights the socio-economic impacts of HIV/AIDS on women. It argues that the socio-cultural beliefs that value the male and female lives differently lead to differential access to health care services. The position of women is exacerbated by their low financial base especially in the rural community where their main source of livelihood, agricultural production does not pay much. But even their active involvement in agricultural production or any other income ventures is hindered when they have to give care to the sick and bedridden friends and relatives. This in itself is a threat to household food security. The paper proposes that gender sensitive policies and programming of intervention at community level would lessen the burden on women who bear the brunt of AIDS as caregivers and livelihood generators at household level. Improvement of medical facilities and quality of services at local dispensaries is seen as feasible since they are in the rural areas. Other interventions should target freeing women's and girls' time for education and involvement in income generating ventures. Two separate data sets from Western Kenya, one being quantitative and another qualitative data have been used.

## Background

Public health services are becoming increasingly important in Kenya, where more than 2.5 million people are living with HIV/AIDS. Currently bed occupancy due to HIV/AIDS is 50–60% in public hospitals. To ease both financial constraints and congestion in the public health facilities, the Kenyan government, following suggestions from the World Bank and other donor agencies, has promoted the introduction of cost sharing in the Kenyan public health facilities and home-based care for HIV/AIDS patients.

## Objectives

This note describes the use of health services and provision of home based care in AIDS stricken communities in Western Kenya and associated social and economic costs.

## Methods

Findings are drawn from two separate data sets from Western Kenya. The first one adds to nation-wide data on 1,422 households in 1997 and revisited in 2000, and 2002. The survey was undertaken by Egerton University's Tegemeo Institute. The survey was undertaken using the agricultural sampling frame of the Central Bureau of Statistics. Between 1997 and 2000, the reinterview rate was 94%, but it fell to 83% between 2000 and 2002. Details on sampling design and data are provided in Yamano and Jayne [1]. From this panel data, households reporting a death in the family between 16–59 years old were revisited in August 2002 and asked about the adjustment processes and behavior of households experiencing recent prime-age mortality. This follow-up survey was conducted in three districts where prime-age mortality was most prevalent: Kisumu, Siaya, and Kakamega districts. Information regarding 31 deceased prime-age adult members was collected in this module.

The second data set is based on in-depth interviews of 25 purposively selected households in 2000. The selection was from the focus group discussions held prior. Findings are based on information regarding hospitalization of 35 deceased members and provision of care to 10 sick members. This method was appropriate because it facilitated the gathering of narratives and experiences related to gender, access to health services and cultural dimensions of HIV/AIDS [2].

Being a policy paper details of research methodology have been omitted to avoid making the paper too long.

## Findings

**First**, regarding the use of medical facilities, we find that, prior to their death, sick adults visited local dispensaries (including public hospitals) and private hospitals regularly (whenever necessary in their view) before they passed away (Table 1). Some of the sick also use services of traditional healers and herbs as well as divine healing where patients are taken for prayers.

**Table 1:** Medical facility use by deceased (15–59 years old) in Western Kenya

Number of deceased		Local Dispensaries			Private Hospitals			Traditional Healers		
		
		Regularly	Occasionally	Once or never	Regularly	Occasionally	Once or never	Regularly	Occasionally	Once or never
		- Percent (s.d.)-			- Percent -			- Percent -		
Male	13	54 (51.9)	23 (42.9)	24 (43.8)	62 (50.6)	15 (37.6)	23 (43.8)	31 (48.0)	23 (43.8)	46 (51.9)
Female	18	72 (46.1)	6 (23.6)	22 (42.8)	56 (51.1)	17 (38.3)	28 (46.1)	44 (51.1)	17 (38.3)	39 (50.2)
Total	31	65 (48.6)	13 (34.1)	23 (42.5)	58 (50.2)	16 (37.4)	26 (44.5)	39 (49.5)	19 (40.2)	42 (50.2)

Source: The Tegemeo Agricultural Monitoring and Policy Analysis Project (TAMPA) Survey collected by Tegemeo Institute/Egerton University.

**Second**, 19 men (some 90%) out of the total of 21 afflicted men received hospitalised care before their death (Table 2). By contrast, only 9 (64%) out of 14 afflicted women received such services before their death.

**Table 2:** Hospitalization of the deceased (19–59 years old) prior to mortality

Gender	Number of deceased	% of total dead	Source of income when alive			Hospitalization^A^	
			
			Waged	Business	Farming/other	Number hospitalized	Number not hospitalized
Male	21	60	10	7	4	19 (90%)	2 (10%)
Female	14	40	4	1	9	9 (64%)	5 (36%)
Total	35	100	14	8	13	28 (80%)	7 (20%)

Note: (A) Stayed at a hospital at least one day.

Source: In-depth interviews with 25 purposively-selected households in Siaya district, interviewed by Opiyo in 2000

Men were more likely to be admitted to hospital multiple times, while most females were admitted only once before death. One frequently given reason for delay for women's hospitalisation was lack of someone to remain with children at home even where the husbands were still alive and well. Further discussions revealed that culturally the lives of men and women are valued differently. This is expressed in sayings such as "*wouyi siro*" (men or boys are pillars). Indeed it is also seen to be easy to replace a wife as men can be polygamous. Thus there tends to be greater efforts to save the life of a male compared to that of a female.

**Third**, Table 2 also shows that men are more involved in off-farm income generation opportunities while women mainly depend on farming for income. Discussions revealed that households afflicted by prime-age sickness sold a number of assets like bicycles, radios and small animals to get more money for medication.

Because women have relatively little cash to use at their own discretion and depend on their husbands' decisions even on issues concerning their health, many women are unable to receive hospital care. But they visit local dispensaries as often as men (Table 1), presumably because medical fees are cheap at local dispensaries and they are familiar with local dispensaries through pre-natal care and childcare.

**Fourth**, women are the primary-care givers to the sick. From Table 3, men did not provide care, except one teenage boy. The women spend active days and nights providing care to bed ridden relatives, coupled with their general domestic chores. Sick females receive care from their female children and female friends. Some of these friends are fellow members in women's guilds while others are relatives, such as aunts, sisters and mothers. In an agricultural economy where women take the largest stake in farming activities there is bound to be serious implications for household food security. On the other hand, it is not socially permissible for women to leave their homes when their husbands are bedridden for whatever reason.

**Table 3:** Provision of home-based care to the adult bedridden patients (19–59 years old)

Gender	Number of sick	Provision of care by:						
		
		Wife	Husband	Mother	Sister	Teenage boys	Teenage girls	Other women
Males	5	2	0	2	2	1	0	0
Females	5	0	0	1	0	0	3	1
Total	10	2	0	3	2	1	3	1

Source: In-depth interviews with 25 purposively-selected households in Siaya district, interviewed by Opiyo in 2000

## Discussions

This paper highlights the importance of considering the economic, social and cultural gender aspects that hinder women from accessing specialized and early health attention even when they need it. This corroborates with other findings in which being a woman and financially incapable has led to inability to access professional health services. Otwombe et al [3] shows that more men than women visit VCT facilities and are therefore more likely to promptly receive the required health care services. Today many VCT centres are found mainly in urban areas and district level. As home based care providers women point out that often the poorly equipped but charging health facilities are also far away with few health providers [4,5].

So long as lives are valued differently there will always be differences in investment in potential life saving activities such as medical treatment and prevention from HIV/AIDS infection. If a woman's life is considered less valuable than a man's, it is likely that when she is ill she will be given or allowed access to fewer care/treatment opportunities. This finding corroborates what Ambasa-Shisanya [6] terms as low premium on lives of women.

The patriarchal nature of the community together with predominant male control of property, general household finances and decision making further exacerbate the position of women and their ability to access specialised treatment and control over their lives. This has been observed to be as a result of low income and education level [6,7]. According to UNICEF report [8], women in sub-Saharan Africa face more discrimination from their spouses, where they have no control over their health care needs. The report observes that women who have greater influence in decision making can promote better health care practices for the family. Though Kenya ratified and subscribes to the Convention on Elimination of all Forms of Discrimination Against Women (CEDAW) very little has been done.

While women provide care, they themselves are often denied this when they need it. They are stigmatised and may be dispossessed and chased from their matrimonial homes especially when they cannot take in cultural rites. They end up depending on their young children for care [9,6,10]. Thus the gender equality and well-being of children go hand in hand.

## Policy Implications

First, we find that even in rural areas, a high proportion of people rely on local dispensaries and hospitals (both public and private) for medical care. Thus, improving medical facility and quality of medical services provided at dispensaries and hospitals will help patients (not only AIDS patients but also other patients) in rural areas. These services should be affordable to all users.

Because patients use traditional healers, their operational conditions should not be downplayed when looking at health structures/policies.

Second, while caring for sick members, women and girls have to reduce their time spent in other activities, such as non-farm self-employment and schooling. As a result, the burden of caregiving leaves women and girls in a weakened economic position, with less money and education. The establishment of functional community based health referral system linked to the mainstream health through community home based care groups would not only support women and men to seek medical attention from better equipped hospitals, it would also at least partially relieve women and girls of the caregiving burden and hence improve their access to livelihood activities and educational opportunities. The financial sustainability of community-based care could be enhanced by public sector support for increased agricultural production and other income-earning opportunities targeted to communities and/or households that have been particularly hard-hit by HIV/AIDS.

Home-base care has direct impact on agricultural production that is mainly done by women in the rural, therefore user-friendly technologies that save time and labor should be developed to seal the gap.

Appropriate women empowerment programs that involve male participation should be supported through enacting gender sensitive health policies. Such a framework would support identification, lobbying and advocacy against socio-cultural beliefs and practices that value the male and female lives differently leading to differential access to health services.

Finally, equal and affordable opportunity in education for girls not only at basic but at higher levels would offer a long term solution as it would increase the worth of the girls/women. Increased numbers of educated, knowledgeable and skilled women would increase their presence in decision making organs. This would spur gender sensitive programming and interventions to lower levels where most women are currently.

## Competing interests

The author(s) declare that they have no competing interests.
